# *in vitro* comparative evaluation of stress distribution around zygomatic versus conventional implants in atrophic maxilla

**DOI:** 10.6026/973206300214527

**Published:** 2025-12-15

**Authors:** Vishwannath Hiremath, Darshan Modi, Kuldip R Panchal, Snigdha Somi, Syed Sajid Basha, Esther Lhingneihoi Mate

**Affiliations:** 1 Department of Oral and Maxillofacial Surgeon, A Unit of Hiremath Hospitals Pvt Ltd) Vijayanagar Bangalore 560040, India; 2 Department of Periodontics, Karnavati school of dentistry, Uvarsad, Gandhinagar, Gujarat, 382422, India; 3 Department of Oral and maxillofacial surgery, Darshan Dental College and Hospital, Loyara, Rajasthan, India; 4 Department of Prosthodontics, Dental Surgery VMMC & Safdurjung New Delhi, India; 5 Department of Oral and Maxillofacial Surgery, Maharana pratap college of dentistry and research centre, Gwalior, India; 6 Kalinga Institute Of Dental Science, KIIT Deemed To Be University, Bhubaneswar, Odisha, India

**Keywords:** Zygomatic implants, finite element analysis, biomechanics, stress distribution, atrophic maxilla, dental implants, bone-implant interface

## Abstract

Severe maxillary atrophy restricts the conventional position of the implants because of insufficient bone height; the alternative anchorage systems like the zygomatic implants should be used. Therefore, it is of interest to compare the distribution of stress under zygomatic and conventional implant designs during simulated occlusive loading. Six maxillary models were tested in the von Mises stress, principal stress and microstrain when the forces are vertical and oblique. Quad-zygomatic arrangements were the least stressed and the most evenly distributed, being far within the range of physiological values. Zygomatic implants, particularly the quad-zygomatic configurations, have better biomechanics in atrophic maxillae than standard methods of implants.

## Background:

Extensive maxillary atrophy caused by long-term edentulism, loss of teeth through trauma, or disease causes significant difficulties in rehabilitation with implants [1]. Conventional modalities of using conventional dental implants in atrophic maxillae usually involve massive bone augmentation surgeries, such as maxillary sinus elevation, onlay bone grafting, or both [2]. Although the methods have the ability to recover bone volume, several methods have been linked with prolonged treatment regimens, morbidity, additional surgical sites and unpredictable graft resorption and high costs [3]. A different form of treatment modality is zygomatic implants, which were introduced by Brånemark in the late 1980s, and which avoids the necessity of bone augmentation by anchoring the implants to the zygomatic bone and not to the remaining maxillary alveolar process [4]. This method utilizes the desirable bone quality and bone volume of the zygoma that is not severely affected by the resorption sequence after losing teeth [5]. Other clinical trials have documented a positive long term survival of zygomatic implants with success rate of over 95 percent after 10 years follow up [6]. Although there is growing clinical evidence to endorse the effectiveness of zygomatic implants, there is a lack of comprehensive insight into the pattern of biomechanical and stress distribution in atrophic maxillaes in which zygomatic implants have been used to restore defects compared to conventional methods [7]. The reason why biomechanical analysis is essential in predicting long-term success of implants is the fact that the focus on excessive stress might cause bone resorption, implant failure, or even complications during the use of a prosthetic [8]. Zygomatic implants have unique geometry, length, angulation, and pattern of anchorage which imply the existence of radically different stress transfer processes in contrast to traditional axial implants [9]. FEA is a potent computing method of stress distribution in complicated biological systems that can be used to thoroughly examine stress distribution that would otherwise be unmeasurable in clinical practice [10]. Past FEA research has investigated zygomatic implants in different configurations, but most of the research has been only on particular loading conditions or limited groups of comparison [11]. Extensive comparative studies on the assessment of various implant designs based on different loading conditions are few within literature [12]. More recent studies have proposed quad-zygomatic design (four zygomatic implants but no traditional implants) as having biomechanical benefits, although there are no direct comparative data between conventional augmentation methods and quad-zygomatic design [13]. Moreover, the allocation of stress across cortical and cancellous bone, strain magnitude in peri-implant tissues, and loading direction effect necessitates a systemic study in the various treatment modalities [14]. There are still critical areas of knowledge deficiency in terms of the best choice of implant configuration biomechanically, patterns of stress distribution during physiological loading conditions, and comparative functions of zygomatic and conventional methods in severely atrophic maxillae [15]. These biomechanical relations are imperative in evidence-based treatment planning and can inform the changes in protocols to maximize long-term results. Thus, this *in vitro* experiment was aimed at a detailed comparison of the stress distribution patterns around zygomatic implants and traditional implants in severely atrophic maxillae with the help of the three-dimensional finite element analysis. The specific objectives were: (1) measurement of von Mises stress, principal stress, and microstrain distributions under different loading conditions; (2) comparison of stress patterns of different implant configurations; (3) measurement of stress distribution in cortical and cancellous bone; (4) determination of the most appropriate biomechanical configurations during atrophic maxilla rehabilitation. Our hypothesis was that zygomatic implant designs would exhibit better stress distributions with lesser maximum stress values than more traditional implant designs.

## Materials and Methods:

## Study design:

This comparative *in vitro* biomechanical study utilized three-dimensional finite element analysis to evaluate stress distribution patterns in six different implant configurations for severely atrophic maxilla rehabilitation.

## Model development and geometric construction:

## Base anatomical model creation:

A severely atrophic edentulous maxilla was reconstructed from computed tomography (CT) data of a 58-year-old patient (anonymized data from institutional database, IRB approval: UMC-IRB-2022-893). The CT scan (Siemens SOMATOM Definition AS+, slice thickness 0.5mm) was imported into medical imaging software (Mimics 21.0, Materialise, Belgium) for segmentation. The atrophic maxilla model exhibited the following characteristics: residual alveolar bone height <5mm in posterior regions, sinus pneumatization extending to within 2-3mm of alveolar crest, average bone width 4-6mm in premolar region, severe bone resorption corresponding to Cawood and Howell Class V-VI. Maxillary bone was segmented into cortical (1-2mm thickness) and cancellous components based on Hounsfield unit thresholds (cortical: >600 HU; cancellous: 150-600 HU). Three-dimensional models were exported as stereolithography (STL) files and imported into computer-aided design software (SolidWorks 2022, Dassault Systemes) for geometric refinement and implant placement planning.

## Implant configuration models:

Six models were developed representing different treatment approaches:

Model 1 (ZI-only): Bilateral zygomatic implants (4.0mm diameter, 52.5mm length, Nobel Biocare Zygomatic implant geometry) placed bilaterally with emergent positions in first molar regions, angulation approximately 45° to occlusal plane.

Model 2 (Quad-ZI): Four zygomatic implants (bilateral anterior: 4.0x42.5mm emerging at canine positions; bilateral posterior: 4.0x52.5mm emerging at second molar positions), representing quad-zygomatic configuration without conventional implants

Model 3 (Conv-Aug): Six conventional implants (4.3mm diameter, 8.5mm length short implants, external hex connection) placed in augmented maxilla simulating bilateral sinus augmentation with 12mm vertical bone gain. Implant positions: bilateral canines, first premolars, and second molars.

Model 4 (ZI-Conv): Combined approach with bilateral zygomatic implants (4.0x52.5mm) in posterior regions plus two anterior conventional implants (4.3x13mm) in augmented anterior maxilla.

Model 5 (Tilted-Conv): Six tilted conventional implants (4.3x13mm) placed in residual bone with posterior implants tilted 30-45° to avoid sinus, following All-on-4 biomechanical principles adapted to maxilla.

Model 6 (Control): Edentulous atrophic maxilla without implants for baseline comparison.

All models included screw-retained full-arch fixed prostheses with identical prosthetic design (metal framework: chrome-cobalt alloy; veneer: acrylic resin), cantilever extension of 10mm distal to most posterior implant.

## Material properties and mesh generation:

Material properties were assigned based on literature values and represented as homogeneous, isotropic, and linearly elastic materials:

[1] Cortical bone: Young's modulus (E) = 13,700 MPa, Poisson's ratio (ν) = 0.30

[2] Cancellous bone: E = 1,370 MPa, ν = 0.30

[3] Zygomatic bone: E = 18,600 MPa, ν = 0.26

[4] Titanium implants (Grade 4): E = 110,000 MPa, ν = 0.35

[5] Chrome-cobalt framework: E = 218,000 MPa, ν = 0.33

[6] Acrylic resin: E = 3,000 MPa, ν = 0.35

Perfect osseointegration was assumed with bonded contact between implant surfaces and bone (contact defined as bonded/tied, no sliding or separation permitted). Framework-implant connections were similarly modeled as bonded. Finite element meshes were generated using ANSYS Workbench 2022 R1 (ANSYS Inc., USA). Tetrahedral elements with 10 nodes (SOLID187) were used throughout. Mesh refinement was applied at implant-bone interfaces and stress concentration areas. Convergence testing determined optimal element size (0.5mm at interfaces, 1.5mm in bulk bone).

Final meshes contained:

[1] ZI-only: 847,562 elements, 1,289,447 nodes

[2] Quad-ZI: 1,124,378 elements, 1,687,293 nodes

[3] Conv-Aug: 923,156 elements, 1,398,562 nodes

[4] ZI-Conv: 982,347 elements, 1,476,891 nodes

[5] Tilted-Conv: 896,234 elements, 1,362,478 nodes

## Boundary conditions and loading scenarios:

The superior and posterior surfaces of maxillary bone and zygomatic bone were constrained in all directions (fixed support), simulating attachment to skull base and cranial structures.

Loading scenarios simulated physiological masticatory forces:

## Vertical loading: Forces applied perpendicular to occlusal plane at three locations:

[1] Anterior region (canine area): 100N

[2] Premolar region: 150N

[3] Molar region: 200N

##  Oblique loading:

Forces applied at 45° angle (30° buccolingually, 15° mesiodistally) representing non-axial chewing forces:

[1] Premolar region: 150N at 45°

[2] Molar region: 200N at 45°

[3] Cantilever region: 100N at 45°

## Combined loading:

Simultaneous bilateral molar loading (150N each side) representing clenching. All loading points were applied on prosthetic occlusal surfaces distributed over 4mm² contact areas to simulate cuspal contact.

## Outcome measures and analysis:

Primary outcome measures:

[1] Von Mises equivalent stress (MPa): Indicator of material failure risk

[2] Maximum principal stress (MPa): Tensile stress, relevant for bone resorption

[3] Minimum principal stress (MPa): Compressive stress

[4] Microstrain (µ): Bone deformation, physiological threshold Z<3000 µ

Stress and strain values were evaluated at:

[1] Implant-bone interface (peri-implant bone within 2mm of implant surface)

[2] Cortical bone (alveolar crest and zygomatic cortex)

[3] Cancellous bone

[4] Implant fixture

[5] Prosthetic framework

## Secondary outcomes:

[1] Stress distribution patterns (visualization and qualitative assessment)

[2] Percentage of bone volume exceeding critical stress thresholds

[3] Location of maximum stress concentration

## Statistical analysis:

Quantitative stress and strain data were extracted from FEA results at predefined nodes of interest (n=500 nodes per model at implant-bone interface; n=1000 nodes in cortical bone; n=1000 nodes in cancellous bone). Descriptive statistics included mean ± standard deviation, median, and range for continuous variables.

One-way analysis of variance (ANOVA) compared mean stress values across the five implant configuration models for each loading scenario, followed by Tukey's post-hoc test for pairwise comparisons. Statistical significance was set at p<0.05. Percentage of bone volume exceeding critical thresholds was calculated and compared using chi-square test. All statistical analyses were performed using SPSS version 28.0 (IBM Corp., USA) and MATLAB R2022b (MathWorks, USA) for data processing.

## Results:

Table 1 (see PDF) presents von Mises stress values in peri-implant bone under different vertical loading scenarios.

Post-hoc comparisons (Tukey's test) for premolar loading (150N):

[1] Quad-ZI vs Conv-Aug: p<0.001

[2] Quad-ZI vs Tilted-Conv: p<0.001

[3] ZI-only vs Conv-Aug: p=0.003

[4] ZI-Conv vs Conv-Aug: p<0.001

[5] Conv-Aug vs Tilted-Conv: p=0.012

Under vertical loading at premolar region (150N), Quad-ZI configuration demonstrated significantly lower von Mises stress (18.4 ± 3.2 MPa) compared to all other configurations except ZI-Conv (22.5 ± 3.8 MPa, p=0.068). Conventional augmentation approach (Conv-Aug) showed 88.6% higher stress than Quad-ZI (p<0.001). Tilted-Conv configuration exhibited the highest stress values (42.3 ± 6.4 MPa), 130% higher than Quad-ZI (p<0.001). At molar loading (200N), stress values increased proportionally across all configurations while maintaining similar relative patterns. ZI-only showed intermediate performance between quad-zygomatic and conventional approaches. Table 2 (see PDF) presents maximum principal stress (tensile) and minimum principal stress (compressive) values under oblique loading conditions.

Statistical comparisons (ANOVA with Tukey's post-hoc):

[1] Maximum principal stress: F(4,2495)=287.4, p<0.001

[2] Quad-ZI vs Conv-Aug: mean difference -27.7 MPa, 95% CI: -31.2 to -24.2, p<0.001

[3] Quad-ZI vs Tilted-Conv: mean difference -36.1 MPa, 95% CI: -40.1 to -32.1, p<0.001

Under oblique loading simulating non-axial masticatory forces, tensile stress (maximum principal stress) was significantly lower in zygomatic configurations compared to conventional approaches. Quad-ZI demonstrated 49.2% lower mean tensile stress than Conv-Aug (p<0.001) and 55.8% lower than Tilted-Conv (p<0.001). Stress concentration patterns differed markedly between configurations. In zygomatic models, peak stresses localized to zygomatic bone in the apical portion of implants, while conventional implant models showed stress concentration at alveolar crest around implant necks. The Quad-ZI configuration exhibited the most distributed stress pattern with lowest peak values (41.2 MPa maximum). Compressive stress (minimum principal stress) followed similar patterns, with zygomatic configurations showing 39.4-46.7% lower compressive stress than conventional approaches (p<0.001). All compressive stress values remained below cortical bone yield strength threshold (approximately -170 MPa). Table 3 (see PDF) presents microstrain distribution and percentage of bone volume exceeding critical physiological thresholds.

Post-hoc pairwise comparisons:

[1] Quad-ZI vs Conv-Aug: χ^2^=89.3, p<0.001

[2] Quad-ZI vs Tilted-Conv: χ^2^=127.6, p<0.001

The microstrain analysis indicated that Quad-ZI configuration retained highest percentage of bone volume at the appropriate physiological strain (50-3000 1 /) and only 2.7 percent of bone volume strain was over 3000 1 /, which is the bone overload and resorption sign of bone overload. On the contrary, Conv-Aug and Tilted-Conv setups had 23.4% and 31.6% of bone volume respectively above this threshold (p<0.001 vs Quad-ZI). Mean cortical bone microstrain in Quad-ZI (1524 ± 298 µe) was kept in an optimal range of bone maintenance and remodeling (1000-2500 ue), and Tilted-Conv was near or above the physiological level (3247 ± 612 ue). Quad-ZI (2368 1455107 times) leads to lower values of peak microstrain relative to Conv-Aug (4872 1845487 times) and Tilted-Conv (5394 2103 1845487 times) (p<0.001). Areas vulnerable to disuse atrophy (strain <50 µe) were minimal in Quad-ZI (8.6% of bone volume) and proposed better chances of load transfer and bone maintenance than traditional arrangements. Biomechanical behaviors could be identified through visual analysis of the patterns of stress distribution. Zygomatic implant designs exhibited stress transfer based mainly on long implant body to zygomatic bone, and avoided atrophic maxillary alveolar process. This led to a greater evenness of stress distribution in bigger volume of bone. Traditional implant designs had a concentration of stress at alveolar crest region especially implant necks such that localized high-stress areas were formed. The implant tilted design exhibited further stress concentration at the bending points of the implants. Combined ZI-Conv arrangement shared stress among zygomatic and anterior standard implants, having intermediate stress results with two load-bearing mechanisms.

## Discussion:

This detailed finite element study shows that zygomatic implant designs, especially quad-zygomatic, have better distributions of biomechanical stress than traditional implants in severely atrophic maxillae. Indicators of a much lower von Mises stress, principal stress and microstrain values in zygomatic structures indicate better biomechanical conditions to preserve bones in the long run, and the success of implants. The tensile stress reduction by 47-49 per cent with quad-zygomatic set up relative to conventional methods has significant biological significance [1. High tensile stress on the bone as the interface with the implant is linked with bone resorption and implant failure [2]. The patterns of stress distributions we have observed in our study where zygomatic configurations absorb load with better quality zygomatic bone instead of the resorved maxillary bone, is consistent with the biological reasoning of using this mode of treatment [3]. The analysis of microstrain gives specific information on the forecasted bone remodeling responses. There is homeostasis in bone in a certain strain threshold (about 50-3000 µ), and excessive strain (>3000 µ) leads to resorptive mechanisms and too little strain (<50 µ) results in disuse atrophy [4]. The fact that the quad-zygomatic set could preserve the bone volume at 88.8% in the range of strains that would be optimum, whereas conventional augmentation would preserve only 56.9% is an indication that the quad-zygomatic configuration had a higher potential of preserving bone in the long-run [5]. This observation can be used to clarify clinical results of a stable bone levels around zygomatic implants during a long follow-up time [6]. The pattern of stress concentration of our research shows inherent differences between the mechanisms of load transfer between zygomatic and conventional methods [7]. Traditional implants on atrophic maxillae focus the stress on the alveolar crest, where the quality and the quantity of bone are already reduced. This mechanical disadvantage is further increased during tilted implants where non-axial loading generates bending moments and high stress levels at the implant necks [8]. By contrast, zygomatic implants are anchored on the strong cortical bone of the zygoma with the use of their apical features to spread stress to a greater mass of superior bone [9]. The exceptional results of the quad-zygomatic structure compared to the bilateral zygomatic structure indicate that more zygomatic implants are beneficial in giving an added biomechanical benefit [10]. The four-implant zygomatic technique forms a larger support polygon and a better balance of forces especially in oblique loading that mimics real chewing patterns. This observation conforms to clinical practice guidelines that recommend quad-zygomatic procedures in the extreme cases of atrophy [11]. The similar or worse outcome of the combined zygomatic-conventional (ZI-Conv) design compared to the quad-zygomatic design was rather surprising since combined designs are typically used in practice [12]. Nonetheless, biomechanical benefit of removing the traditional implants in intense atrophic bone seems to be superior to other benefits of implant support. This insinuates that where there is extreme atrophy, it is biomechanically better to use zygomatic implants to the maximum instead of working with impoverished traditional implant sites [13]. The tilted standard implant design had the worst biomechanical behaviour on all outcome measures, although All-on-4 and similar ideas have been clinical successful in the literature [14]. This perceived inconsistency could be due to variations in mandibular applications (where All-on-4 is mostly used) and maxillary structure, and the extreme atrophy represented in this paper. Increased levels of stress and large areas of bones that are above physiological levels of stress indicate that tilted conventional implants can be more stressful in severely atrophic maxillae than zygomatic implants [15]. A number of significant clinical implications are as a result of these findings. The first reason is in a pure biomechanical sense, quad-zygomatic is an ideal solution in cases of severely atrophic maxillae, giving superior stress distribution to traditional methods such as that of bone augmentation. Second, the fact that transfer of stress occurs to zygomatic bone as opposed to atrophic maxillary bone indicates that the zygomatic implants could be especially beneficial in situations with very poor quality of the bone where the traditional implants would be overloaded. Third, the reduced volume of bones at risk of disuse atrophy with zygomatic designs indicates that stress shielding concerns with zygomatic designs may be unfounded. There are serious limitations that should be mentioned. To begin with, this FEA experiment applied simplified conditions such as homogeneous and isotropic material characteristics, Osseointegration that was ideal and linear elastic behaviour. Real bones are anisotropic, not homogeneous in mineralizing, as well as in viscoelastic behavior. Second, the difficult comparative information is not entirely represented by the static loading conditions typical of mastication, but still is of great value in comparison. Third, the single anatomical model is representative of extreme atrophy, but might not represent complete anatomical variability among patients. Fourth, augmented bone was made with properties that were as strong as native bone, which could be overstating the loading capacity of augmented bone. Fifth, early healing interfaces and micro movements were not modelled using soft tissues. These limitations should be overcome by future studies employing dynamic FEA with cyclic loading, patient-specific modelling of different anatomies, experimental validation with physical models or animal experiments, analysis of the early healing phase with unfinished bone integration, and clinical correlation studies to the prediction of FEA to long-term results. Also, the assessment of prosthetic complications, screw loosening and framework stress would offer full biomechanical evaluation.

## Conclusion:

Advanced design of zygomatic implant with specially designed quad-zygomatic, offer better distribution of biomechanical stress than traditional implants in severely atrophic maxillae. They can distribute loads effectively to dense zygomatic bone reducing the stress on weak maxillary structures. The findings justify zygomatic implants as the first-line treatment option in severe maxillary atrophy and they need to be supported by clinical evidence.
